# On the Mechanical Response of Silicon Dioxide Nanofiller Concentration on Fused Filament Fabrication 3D Printed Isotactic Polypropylene Nanocomposites

**DOI:** 10.3390/polym13122029

**Published:** 2021-06-21

**Authors:** Nectarios Vidakis, Markos Petousis, Emmanouil Velidakis, Lazaros Tzounis, Nikolaos Mountakis, Apostolos Korlos, Peder Erik Fischer-Griffiths, Sotirios Grammatikos

**Affiliations:** 1 Mechanical Engineering Department, Hellenic Mediterranean University, 71410 Heraklion, Greece; vidakis@hmu.gr (N.V.); mvelidakis@hmu.gr (E.V.); mh90@edu.hmu.gr (N.M.); 2 Department of Materials Science and Engineering, University of Ioannina, 45110 Ioannina, Greece; latzounis@uoi.gr; 3 Department of Industrial Engineering and Management, International Hellenic University, 14th Km Thessaloniki—N. Moudania, Thermi, 57001 Thessaloniki, Greece; apkorlos@ihu.gr; 4 Laboratory of Advanced and Sustainable Engineering Materials (ASEMlab), Department of Manufacturing & Civil Engineering, NTNU-Norwegian University of Science and Technology, Building B’ Teknologivegen 22, 2815 Gjøvik, Norway; pederef@stud.ntnu.no (P.E.F.-G.); sotirios.grammatikos@ntnu.no (S.G.)

**Keywords:** additive manufacturing (AM), three-dimensional (3D) printing, nanocomposites, polypropylene (PP), silicon dioxide (SiO_2_), tensile test, flexural test, Charpy impact test, Vickers microhardness, scanning electron microscopy (SEM)

## Abstract

Utilization of advanced engineering thermoplastic materials in fused filament fabrication (FFF) 3D printing process is critical in expanding additive manufacturing (AM) applications. Polypropylene (PP) is a widely used thermoplastic material, while silicon dioxide (SiO_2_) nanoparticles (NPs), which can be found in many living organisms, are commonly employed as fillers in polymers to improve their mechanical properties and processability. In this work, PP/SiO_2_ nanocomposite filaments at various concentrations were developed following a melt mixing extrusion process, and used for FFF 3D printing of specimens’ characterization according to international standards. Tensile, flexural, impact, microhardness, and dynamic mechanical analysis (DMA) tests were conducted to determine the effect of the nanofiller loading on the mechanical and viscoelastic properties of the polymer matrix. Scanning electron microscopy (SEM), Raman spectroscopy and atomic force microscopy (AFM) were performed for microstructural analysis, and finally melt flow index (MFI) tests were conducted to assess the melt rheological properties. An improvement in the mechanical performance was observed for silica loading up to 2.0 wt.%, while 4.0 wt.% was a potential threshold revealing processability challenges. Overall, PP/SiO_2_ nanocomposites could be ideal candidates for advanced 3D printing engineering applications towards structural plastic components with enhanced mechanical performance.

## 1. Introduction

Additive manufacturing (AM) has significantly expanded during the last decade, while research and development on AM technologies herald a promising future [1]. Fused filament fabrication (FFF) three-dimensional printing (3D printing) is one of the AM technologies that is currently widely employed, among others, in applications ranging from home use, up to prototyping and industrial manufacturing on a small scale [2]. Consumer products, operational parts in wide range of machinery and applications, biomedical equipment, etc. are some of the applications where FFF is utilized [3,4,5]. AM provides new opportunities, especially when small batch fabrication is the target. A further feature of FFF technology is the ability to operate almost autonomously even up to 24 h per day and with little requirements regarding tools. This asset also strengthens the ability of FFF equipment to operate even in non-industrial environments, leading to a more decentralized and flexible future production model [3].

FFF manufacturing technology is part of the three-Dimensional printing (3D printing) technologies [6], which are replacing conventional manufacturing through AM, although this is still a hard and challenging task. Currently, there is a lot of research being carried out to identify the necessary specifications in order to replace conventional manufacturing technologies, such as injection molding, thermoforming, machining, and others [7]. 3D printed parts have the advantage to be manufactured with a great geometrical freedom [8]. Additionally, tools’ cost is annihilated through AM utilization [9], while fully tailored made products up to consumer’s demand can be fabricated with AM [10]. In contrast, AM technologies operating principle, which is outlined as a layer-by-layer material deposition, controlled through a computer program (gcode), is responsible for the 3D printed part anisotropic properties, compared to parts that have been manufactured through traditional methods [11]. This anisotropy in the 3D printed parts, is mainly caused by poor interlayer fusion.

Moreover, materials used for AM technologies are produced with properties aiming to improve processability [6]. Most of the existing stock filament materials have nowadays already been thoroughly studied in literature [12,13,14]. To achieve multi-functional 3D printed objects via FFF i.e., exhibiting enhanced mechanical and thermal (or electrical) properties, etc., polymer nanocomposites are considered as ideal candidates [15,16]. However, they may induce some processability issues to the FFF 3D melt filamentous printing process. This is due to the well-known abrupt increase of the polymer melt viscosity, associated with the nanoparticle inclusions hindering the polymer chain mobility in the melt state, which may result in a deteriorated quality of the 3D printed final objects [17,18,19,20]. It is of great scientific interest thus to develop polymer (nano-)composites able to be processed through AM technologies without compromising mechanical, thermal and other inherent properties of the matrix host material [6,17,18,19,20,21,22,23].

Polypropylene (PP) is one of the most widely used thermoplastic polymers in a great variety of engineering applications [24]. High quality mechanical performance and heat resistance have given a standout position of PP in industrial applications [25]. The semi-crystalline nature of PP is the main reason for deviations in the material’s properties, known to be significantly affected by the manufacturing process followed i.e., in melt mixing extrusion processes PP is very “process” sensitive to plausible shear induced crystallization phenomena that may take place during processing [26]. The processing temperature can have also a prominent effect on the crystallization of PP. This is critical to be considered especially for 3D printing, since melt processing temperatures, that may induce strong crystallization of the extruded material, could result into shrinkage phenomena, not desirable for high quality 3D printed objects [27]. Due to this process temperature sensitivity behavior, PP is a hard-to-process material in FFF. High shrinkage and thermal instability create intense wrap phenomena when PP is 3D printed with the FFF process. Such phenomena are responsible for most parts manufacturing failures, tolerance deviations and geometrical inconsistencies. As such, it is of great interest to develop PP formulations that can overcome such processing issues, by increasing thermal stability, to expand the usage of the PP in AM applications.

Silicon dioxide (SiO_2_) commonly referred to as silica, can be found in many living organisms [28]. Sand is mainly composed from quartz-based silica, and its contribution in concrete industry is widely known [19,29]. Silicon dioxide is also utilized as filler in polymer-based composites [30,31,32,33]. Targeting to increase microwave absorption, antimicrobial performance, mechanical properties, etc., silica can be used as a dispersed phase in polymeric materials [20,34]. In many cases SiO_2_ is also accrued as to improve processability of polymers in different manufacturing methods [20,35].

In the current study, the effect of compounding isotactic PP (i-PP) with SiO_2_ NPs, as a filler in low concentrations, on the mechanical, viscoelastic, physicochemical and microstructural properties of the developed nanocomposites, was studied. As such, SiO_2_ at 0.5 wt.%, 1.0 wt.%, 2.0 wt.% and 4.0 wt.% filler loading was dispersed in PP, with a view to studying the sensitivity of filler fraction to PP properties and also determining a potential threshold of filler loading at which PP properties start to degrade. The mechanical performance of 3D printed specimens was evaluated through tensile, flexural, impact and microhardness tests, while viscoelastic thermomechanical properties were evaluated through dynamic mechanical analysis (DMA). No similar has been presented in literature so far. According to literature, although both the matrix material and the filler are popular in industrial applications, there is no similar research available yet for this specific nanocomposite studied in this work for 3D printing, which is a very popular process nowadays in research and in industry, as mentioned above. Additionally, nanocomposites were fully characterized for their mechanical, thermal and rheological properties, according to international standards, ensuring the completeness and the reliability of the presented results, which are analyzed and discussed in detail further below in the study.

Microstructural analysis of the 3D printed samples’ outer surface and internal structure was performed via scanning electron microscopy (SEM), while atomic force microscopy (AFM) was performed onto the extruded filaments revealing the surface topography with respect to the different SiO_2_ NP filler loadings. Raman spectroscopy proved the expected fingerprints of the PP/SiO_2_ nanocomposite materials, whilst rheological investigations revealing the effects of filler loading on the PP melt viscosity were performed via melt flow index (MFI) analyses. Results indicated significant reinforcement mechanisms of the PP/SiO_2_ nanocomposite, even at low fractions, rendering the proposed formulations as promising feedstock for potential industrial-scale FFF 3D printing applications where PP enhanced static mechanical and dynamic thermomechanical properties are required.

## 2. Materials and Methods

### 2.1. Materials

Isotactic polypropylene (i-PP) to be used as the matrix material in the current study sold under the Ecolen PP trademark was procured from Hellenic Petroleum S.A. (Hellenic Petroleum S.A., Athens, Greece). PP was procured in coarse powder form, to improve compounding. Silicon dioxide (SiO_2_) nanoparticles were procured from Sigma-Aldrich Chemie GmbH (Sigma-Aldrich Chemie GmbH, St. Louis, MO, USA), in spherical shape with diameters varying from 5 to 15 nm. The purity of the SiO_2_ powder was over 99.5%. Nanocomposite materials were fabricated in this work in four (4) different SiO_2_ filler concentrations (0.5 wt.%, 1.0 wt.%, 2.0 wt.% and 4.0 wt.%) and their properties were compared to pure PP.

### 2.2. Methods

**Figure 1:**
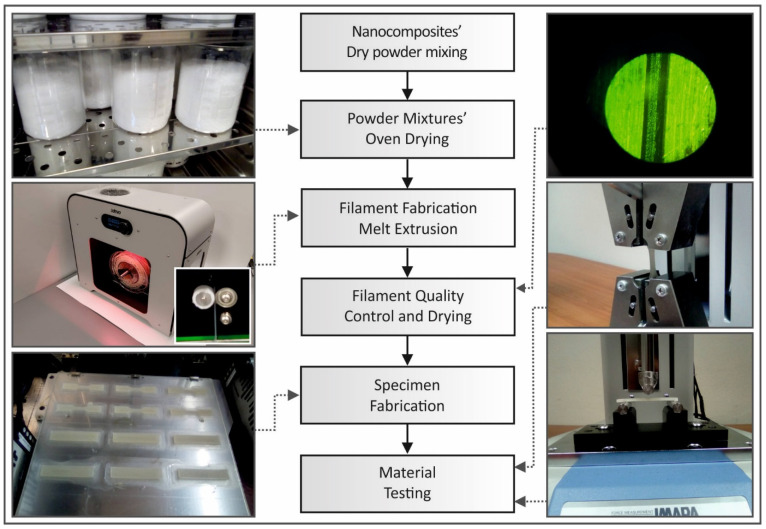
Schematic illustration and photos captured during the processes followed in this study.

A presentation of the followed steps in this work is schematically illustrated in Figure 1. Each step and sub-step followed during this study are described in detail in the next sections.

#### 2.2.1. Filament Production Process

The following process parameters were employed for the preparation of all samples in the current study. Before starting the mixing process, pure PP in coarse powder form was dried at 80 °C for 24 h, using a laboratory oven. After drying, mixing with silicon dioxide (wt.%), for the four filler fractions of this study, followed. PP powder mixing was performed in a closed chamber, using a mechanical mixer to minimize material losses during blending, which could result to a change in filler percentage. Nanocomposites and pure PP powders were further dried afterwards at 80 °C for 4 h, to reduce moisture in the mixing compound.

Filament fabrication of diameter suitable for 3D printing (1.75 mm) followed, using a 3D Evo Composer 450 (3D Evo B.V., Utrecht, The Netherlands) extruder. Composer 450 is a single screw extruder designed for the optimization of the melt-mixing process. It is equipped with a four (4) heating zones chamber and a built-in real-time filament diameter measurement system. In this way, the rotational speed of the winder is automatically adjusted in real time during the extruding process, according to the filament’s measured diameter, to minimize the produced filament diameter deviations.

Heating zone 4 (closer to hopper) was set at 195 °C, heating zone 2 and 3 (middle stage) at 210 °C and finally heating zone 1 (closer to extruder’s nozzle) at 205 °C. Extruder’s screw rotational speed was adjusted to 3.5 rpm. The cooling system of the extruder was set to 40%, while an external duct was utilized in order to achieve a smooth cooling process. This duct was designed to drive air around the extruded material, aiming to improve filament’s roundness. This feature was experimentally found to be necessary for the specific materials of this work. Quality control was performed using both the built-in measurement system, with measurements recorded in real time, and manually, using a high-quality caliper. The achieved filament diameter was 1.75 ± 0.07mm throughout the filaments’ extrusion process.

#### 2.2.2. Tensile Specimens Fabrication and Testing

**Figure 2:**
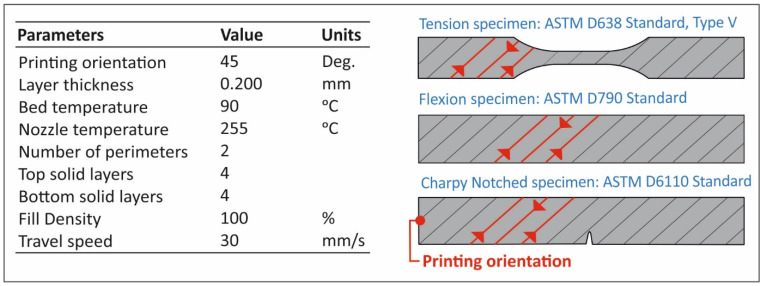
Settings employed in the additive manufacturing FFF 3D printing process for specimens’ fabrication in this work.

An Intamsys Funmat HT (Intamsys Technology ltd, Shanghai, China) was utilized for the 3D printing process of the specimens. A 0.4 mm diameter nozzle was used for all materials in a closed chamber 3D printer to maintain constant temperature conditions and minimize the “wrapping” effect on the 3D printed specimens. The gcode required for the 3D printing process was prepared using the Intamsuite software (Intamsys Technology Ltd., Shanghai, China), with the settings shown in Figure 2. All the other settings for the 3D printing process (not referred in Figure 2) were set to the default value of the Intamsuite software, using PP as the selected material.

Tensile specimens were fabricated following the ASTM D638-02a international standard. Five (5) specimens were 3D printed for all the materials tested. ASTM D638-02a standard type V specimens with 3.2 mm thickness were manufactured. The tensile apparatus utilized for the tests was an Imada MX2 system (Imada Inc., Northbrook, IL, USA). Test rate was set to 10 mm/min under room temperature conditions (22 °C and 50% RH).

#### 2.2.3. Flexural Specimens Fabrication and Testing

Flexural specimens were manufactured following the same process described in Section 2.2.2. ASTM D790-10 international standard was followed for specimen’s testing. According to the standard, five (5) specimens of 3.2 mm thickness were fabricated for each material studied, so a total of twenty-five (25) flexural specimens were manufactured and tested. Flexural tests were conducted using also the Imada MX2 apparatus, in three-point bending setup (bottom-right picture of Figure 1). Test rate was adjusted to 10 mm/min and tests were conducted at room temperature conditions (22 °C and 50% RH).

#### 2.2.4. Charpy Impact Specimens Fabrication and Testing

ASTM D6110-04 standard was followed for notched specimens’ Charpy Impact tests. According to the standard, specimen dimensions were 80 mm length, 10 mm width and 8 mm height. Specimens were fabricated using the process described in Section 2.2.2. For impact testing, a Terco MT220 Charpy (Terco AB, Kungens Kurva, Sweden) apparatus was utilized. A total of 25 specimens were tested (five for each material case), employing release height of 367 mm at room temperature conditions (22 °C and 50% RH).

#### 2.2.5. Microhardness Measurements

Microhardness is related to the plasticity of the material, which is directly connected with the material mechanical response [36]. Vickers microhardness measurements were carried out at randomly selected specimens produced for tensile, flexural or impact testing. Specimens’ surfaces were thoroughly polished prior to taking measurement, to minimize roughness due to the 3D printing manufacturing process. Microhardness measurements were taken with an Innova Test 300-Vickers laboratory machine (Innovatest Europe B.V., Maastricht, The Netherlands). Indentations were conducted by setting the applied force at 300 gF, while indentations’ duration was set to 10 s. Each material’s microhardness was calculated as an average of five (5) measurements, taken at temperature of 22 °C and ~50% RH.

#### 2.2.6. Dynamic Mechanical Analysis (DMA)

DMA specimens were fabricated using the 3D printing process described at Section 2.2.2. Batches of three (3) specimens were manufactured and tested for each material case. DMA was conducted on a TA Instruments DMA850 (New Castle, DE, USA) apparatus. Dimensions of the 3D printed specimens were of 60 mm length, 15 mm width and 3.0–3.2 mm thickness. Prior to testing, all specimens were polished using 200 and 400 grain sandpapers, to smoothen rough side edges from manufacturing process, followed by drying for 48 h at 30 °C. The DMA testing process consisted of a temperature ramp from room temperature to 130 °C, following a rate of 3 °C/min. Test was conducted using a 3-point bending fixture (preload of 0.1 N and 1 Hz frequency). Data was collected at a sampling rate of 0.33 Hz. The recorded parameters are the storage modulus, loss modulus, tan δ, temperature, time, and oscillation angular frequency.

#### 2.2.7. Spectroscopic and Morphological Characterization

Raman spectroscopy was performed for the pure PP, as well as the PP/SiO_2_ nanocomposite 3D printed specimens using a Labram HR-Horiba (Horiba Co Ltd., Kyoto, Japan) scientific micro-Raman system. An optical microscope equipped with a 50× long working distance objective was utilized both for delivering the excitation light, as well as collecting the back-scattering Raman activity. An Ar^+^ ion laser line (514.5 nm) at 1.5 mW power at the focal plane was utilized for the Raman excitation. All acquired and depicted spectra in this work have been treated with a baseline correction through subtraction of a linear or polynomial fit of the baseline from the raw spectra in order to remove tilted baseline variation caused by various noises, i.e., fluorescent background, etc.

In order to acquire more information for the microstructural characterization, Scanning electron microscopy (SEM) was performed on a JEOL JSM 6362LV (JEOL Ltd., Peabody, MA, USA) electron microscope in high-vacuum mode at 20 kV acceleration voltage on sputtered-gold coated samples. SEM images were captured from tensile test specimens for all nanomaterials fabricated in this work. Captures were taken to the side and the fracture area of the tensile specimens at various zoom levels.

Atomic force microscopy (AFM) was also utilized for the morphological characterization of the produced filaments of this work. A MicroscopeSolver P47H Pro NT-MDT (Moscow, Russia) was utilized for the AFM measurements. Filaments’ surface topology was studied at room temperature of 22 °C, with a resonant frequency of about 300 kHz. Silicon cantilevers available at the market were used with scanning frequency of 1 Hz and the remaining parameters for the measurements were as follows: cantilever spring constant was 35 N/m, the tip cone angle was 20° and the tip radius was approximately 10 nm.

#### 2.2.8. Melt Flow Index

Melt flow index tests were conducted following the ISO 1133:2005 international standard. A CEAST MF20 Melt Flow Tester (Instron, Norwood, MA, USA) was utilized for the purposes of the current study. PP and the four nanocomposites fabricated in this work were tested for their ease of flow in the melt state. The basic principle of the method involves the thermal heating (230 °C in this study) of a granulated thermoplastic to molten state and its forced flow out of a capillary die. An extruding piston is used that is loaded with dead weights, up to 21.6 kg. In this work, 4 min pre-conditioning (in the chamber) without any weight was employed, using dead-weights of 2.16 kg, at room temperature conditions. Melt mass-flow rate (MFR) corresponds to the mass flow an alternative quantity is the volume flow, called melt volume-flow rate (MVR), as expressed in Equation (1):

(1) $$\mathrm{MVR}=\frac{\mathrm{MFR}}{\rho }$$

where, ρ = the polymer’s density in the melt state.

## 3. Results

### 3.1. Mechanical Performance

#### 3.1.1. Tensile Test Results

**Figure 3:**
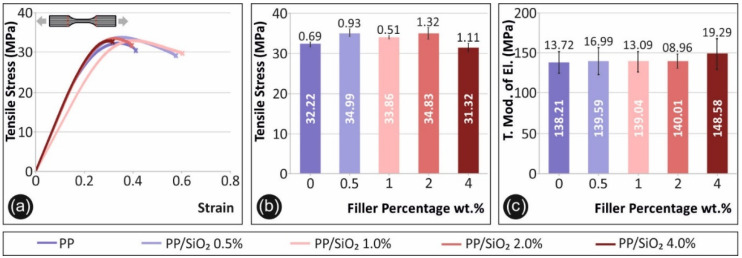
Tensile test results: (**a**) tensile stress (MPa) to strain (%) graph, for a characteristic specimen of each material fabricated, (**b**) average maximum tensile stress (MPa) and its deviation for the materials tested, (**c**) average tensile modulus of elasticity (MPa) and its deviation for the materials tested.

Figure 3 shows results obtained during the tensile tests. Specifically, Figure 3a shows a typical tensile stress (MPa) to corresponding strain graph for each material case tested. Average tensile strength and the corresponding standard deviation of the average value of each material tested is shown in Figure 3b. As shown in Figure 3b, an approximately 10% increase is observed when comparing pure PP maximum tensile stress values to the corresponding values for filler ratios of 0.5 wt.% and 2.0 wt.%. A similar percentage increase is also observed for the average tensile modulus of elasticity, in this case for PP-SiO_2_ 4.0 wt.%, when compared to neat PP (Figure 3c).

#### 3.1.2. Flexural Test Results

**Figure 4:**
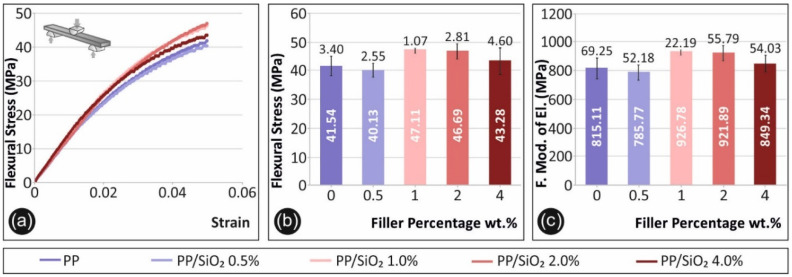
Flexural test results: test results: (**a**) flexural stress (MPa) to strain (%) graph, for a characteristic specimen of each material fabricated, (**b**) average maximum flexural stress (MPa) and its deviation for the materials tested, (**c**) average flexural modulus of elasticity (MPa) and its deviation for the materials tested.

Figure 4 shows results obtained during the flexural tests. Specifically, Figure 4a shows a typical flexural stress-strain graph for each material case tested. As shown, maximum strain for all materials was terminated at 5.0% strain, according to ASTM D790-10. Figure 4b shows the average maximum flexural stress values at 5.0% strain for all tested material cases in the current study.

Figure 4c shows the average flexural modulus of elasticity calculated for each material case, respectively. An increase of ~13%, ~12% and ~4 % is observed for the flexural strength for the cases of PP/SiO_2_ 1.0 wt.%, 2.0 wt.% and 4.0 wt.%, compared to pure PP. A similar trend was observed for the average flexural modulus of elasticity values presented in Figure 3c.

#### 3.1.3. Impact and Microhardness Test Results

**Figure 5:**
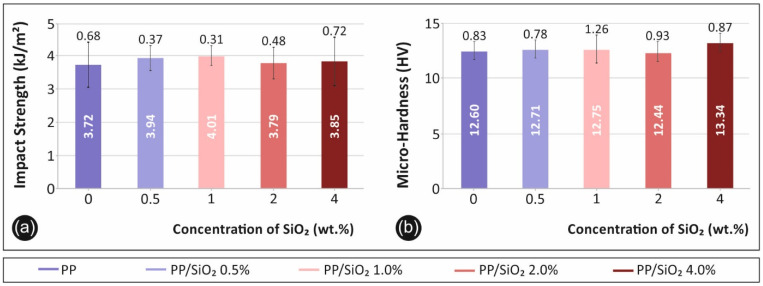
Impact and microhardness test: (**a**) average impact strength (kJ/m^2^) and its deviation and, (**b**) average microhardness (HV) measurements and their deviations for all materials fabricated in this work.

Impact and Vickers microhardness tests results are summarized in Figure 5. Figure 5a depicts the average recorded impact strength values (kJ/m^2^), while Figure 5b presents the Vickers microhardness (HV) average values, for the different PP/SiO_2_ nanocomposite filler loadings. An increasing trend in the average impact strength values was recorded with the increase of the filler loading. However, due to the large experimental scatter, the effect is insignificant. A similar, but of lower magnitude, trend, was observed for the effect of the filler loading on the microhardness values.

### 3.2. DMA Results

**Figure 6:**
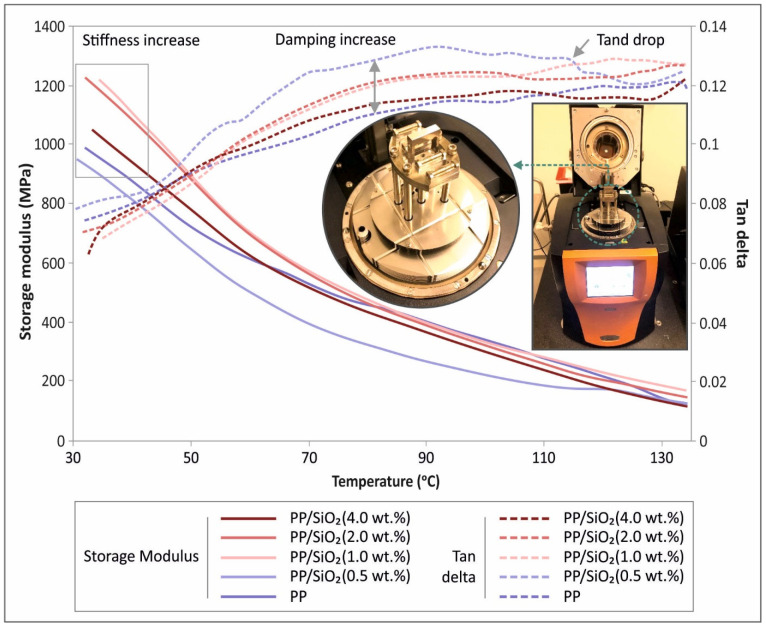
Storage modulus (MPa) left Y axis, and tanδ right Y axis in comparison to DMA’s temperature range for all fabricated materials.

Storage modulus and tanδ as a function of temperature are shown in Figure 6. A general drop of the storage modulus with increasing temperature is observed for all studied materials, that changes its gradient after ~60 °C. Silica presence in ratios higher than 1.0 wt.% shows a stiffening effect on the nanocomposite materials, apart from the case of 4.0 wt.%, which coincides with the findings from tensile and flexure testing. At the same time, damping increased, with filler loading, with 0.5 wt. % exhibiting the most pronounced increase. It is interesting to note that tanδ curves exhibit a general increasing tendency, that saturates after approximately 70 °C, while beyond 110 °C, a oscillating trend is observed, that corresponds to softening at high temperatures, which was more evident for 0.5 wt.%. The latter is in agreement with the tensile and flexure responses due to filler loading (Figure 3 and Figure 4).

### 3.3. Spectroscopic and Microstructural Analysis

**Figure 7:**
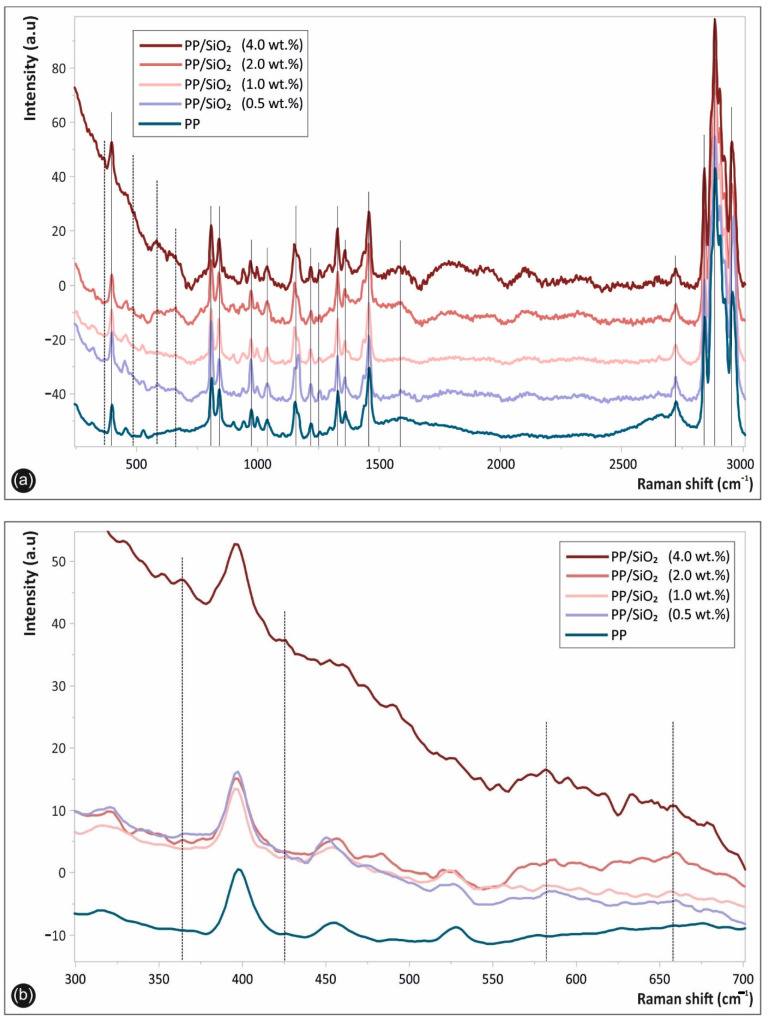
Raman spectra of 3D printed samples, namely the pure PP, as well as the PP/SiO_2_ nanocomposites at different filler loadings, in the spectral region of (**a**) 250–3000 cm^−1^, as well as (**b**) 300–700 cm^−1^, respectively.

Figure 7 shows the Raman spectra of the respective 3D printed samples, namely the pure PP, as well as the PP/SiO_2_ nanocomposites at different filler loadings. Specifically, Figure 7a shows the whole acquired Raman spectrum in the spectral range of 250–3000 cm^−1^, while Figure 7b in the 300–700 cm^−1^ regions. All peaks attributed to the PP matrix macromolecular chains chemistry i.e., due to the polymer chain backbone and the side groups, are depicted with continuous lines, while the specific bands assigned to SiO_2_ NPs are illustrated with dashed lines (Figure 7a,b).

**Figure 8:**
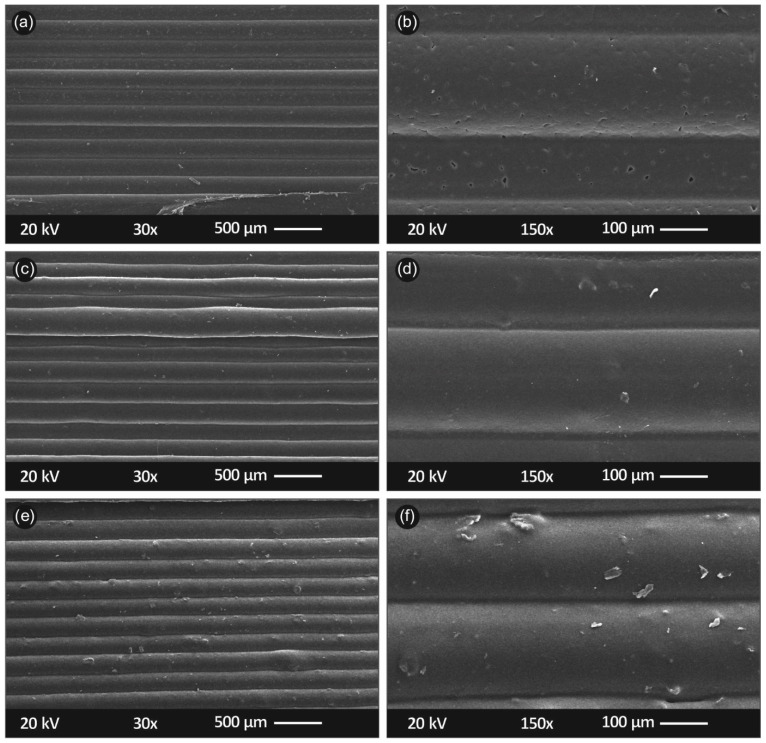
SEM images of the tensile specimens’ side/outer surfaces: (**a**) ×30 magnification of PP/SiO_2_ 0.5 wt.%; (**b**) ×150 magnification of PP/SiO_2_ 0.5 wt.%; (**c**) ×30 magnification of PP/SiO_2_ 2.0 wt.%; (**d**) ×150 magnification of PP/SiO_2_ 2.0 wt.%; (**e**) ×30 magnification of PP/SiO_2_ 4.0 wt.%; (**f**) ×150 magnification of PP/SiO_2_ 4.0 wt.%.

**Figure 9:**
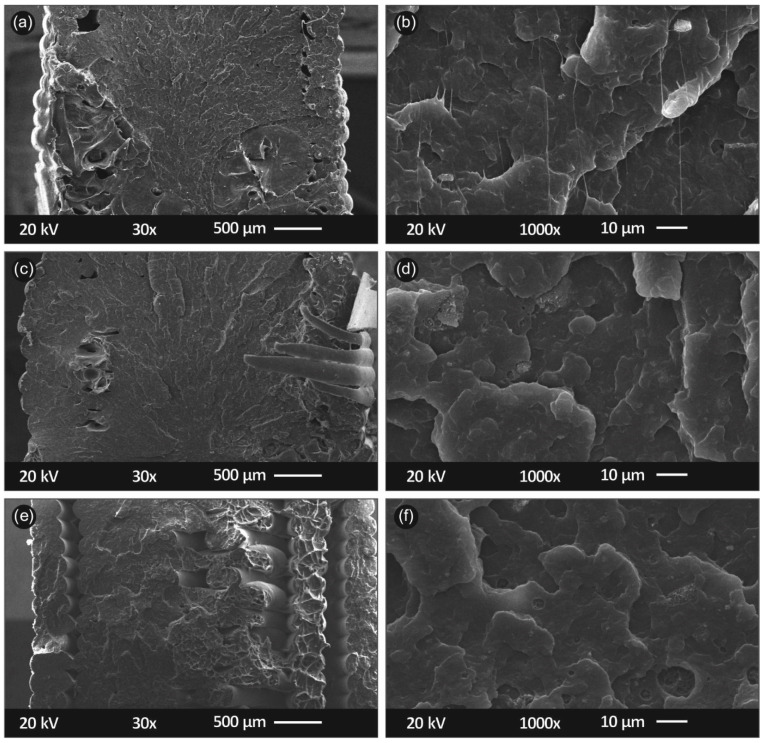
SEM images of the tensile specimens’ fractured areas: (**a**) ×30 magnification of PP/SiO_2_ 0.5 wt.%; (**b**) ×1000 magnification of PP/SiO_2_ 0.5 wt.%; (**c**) ×30 magnification of PP/SiO_2_ 2.0 wt.%; (**d**) ×1000 magnification of PP/SiO_2_ 2.0 wt.%; (**e**) ×30 magnification of PP/SiO_2_ 4.0 wt.%; (**f**) ×1000 mag nification of PP/SiO_2_ 4.0 wt.%.

Figure 8 shows SEM pictures acquired from the side of the tensile specimens. Figure 8a,b show a specimen’s side at ×30 and ×150 magnification respectively, for PP/SiO_2_ 0.5 wt.%. Figure 8c,d show same magnification, in correspondence to Figure 8a,b, for PP/SiO_2_ 2.0 wt.% while Figure 8e,f, for PP/SiO_2_ 4.0 wt.%, respectively. In Figure 9 SEM images captured through the fractured area of the tensile specimens are shown. Specifically, Figure 9a,b correspond to the fractured area of PP/SiO_2_ 0.5 wt.% at ×30 and ×1000 magnification, respectively. Figure 9c,d show the fractured area of PP/SiO_2_ 2.0 wt.%, while Figure 9e,f of PP/SiO_2_ 4.0 wt.%, respectively.

**Figure 10:**
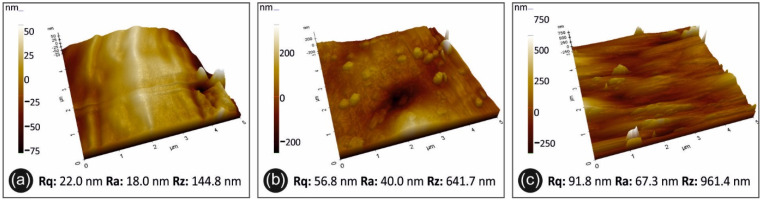
AFM images of the filaments’ side surface for the different materials in this study: (**a**) PP/SiO_2_ 0.5 wt.%, (**b**) PP/SiO_2_ 2.0 wt.%, (**c**) PP/SiO_2_ 4.0 wt.%.

AFM 3D height images are shown in Figure 10. The filament surface topology of PP/SiO_2_ 0.5 wt.% is shown in Figure 10a, and Figure 10b,c correspond to PP/SiO_2_ nanocomposites with 2.0 wt.% and 4.0 wt.% filler loading, respectively. In general, filler concentration has a minor effect in the materials’ microstructure, with further analysis to follow in the next sections.

### 3.4. Melt Flow Index Results

**Figure 11:**
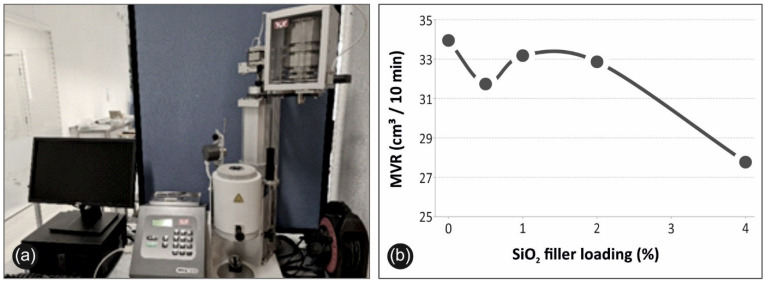
(**a**) Typical setup and equipment used for the MFI analysis, (**b**) MVR in cm^3^/10 min to silicon dioxide ratio (%) in nanocomposite.

Figure 11 illustrates the MFI setup (Figure 11a) and the effect of the filler loading to MVR (Figure 11b). MVR as stated, is a quality control index to determine the ease of flow of each material. Therefore, MVR is one of the parameters related to the processability of the material, which is of prior importance for FFF 3D printing. Silica nanoparticles induced a general drop in MVR, which was an expected result as polymer melt viscosity increases with filler ratio. The highest MVR value was recorded for the case of pure PP (~34 cm^3^/10 min), while the lowest MVR value for the case of PP/SiO_2_ 4.0 wt% (~27.5 cm^3^/10 min).

## 4. Discussion

### 4.1. Mechanical Properties Analysis

With regards to the tensile property response to filler loading, a clear increasing effect was exhibited for the case of the tensile strength of 0.5 wt.%, 1.0 wt.% and 2.0 wt.% (Figure 3b). From Figure 3c, the tensile modulus of elasticity (which is related to the material stiffness) indicates a marginal increasing tendency, which is more pronounced for the case of PP/SiO_2_ 4.0 wt.% (~7% increase compared to pure PP). However, the average value increase is within the experimental scatter, therefore, no significant conclusion can be drawn. Tensile response agrees with literature [37], where an increase of the tensile strength with the increase of silica loading has been reported for non-3D printed specimens. Tensile stiffness increasing tendency leads to an expected brittle behavior [38], which is more prominent in the work, for 4.0 wt.% filler loading. Kruenate et al. [38] reported a 20% reduction in the strain at break for PP/SiO_2_ 2.0 wt.% nanocomposites (compared to pure PP), which coincides with the finding of this this work, for both the 2.0 wt.% and 4.0 wt.% nanocomposites (Figure 3a).

A similar effect to the tensile response was observed for the flexural properties. The increase of silica ratio inclusion within PP leads to an increase of the flexural strength and stiffness. More specifically, flexural properties after revealing a marginal drop for 0.5 wt.% filler loading, improved for the cases of 1.0 wt.%, 2.0 wt.% and 4.0 wt.%, respectively. Interestingly, flexural strength and stiffness exhibited peak values for 1.0 wt.% filler loading that started to diminish with higher loadings. Impact strength and microhardness measurements were insignificantly affected by the introduction of silica to the matrix material.

**Figure 12:**
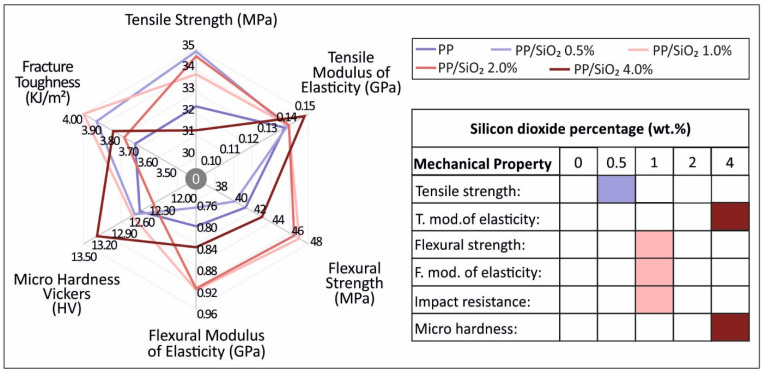
Summary of the mechanical properties measured and/or calculated through the conducted tests. Highest values marked at bottom right side for each material tested for the purposes of current study.

Figure 12 and Table 1 summarize the mechanical response to filler loading. Highest recorded or calculated values are marked on the right side of Figure 12. The 1 wt.% concentration had the better mechanical performance overall among the concentrations studied, showing that even low concentrations are adequate to enhance the response of the material. At 4 wt.% the mechanical response of the material started to decrease, with the average tensile strength of the 4 wt.% specimens studied being marginally lower than the pure PP material, indicating a threshold in the filler loading at this concentration.

### 4.2. DMA Analysis

Storage modulus at low temperatures corresponds to flexural stiffness. As it can be seen from Figure 6, all apart from the 0.5 wt.% filler loading case, resulted into higher stiffness than the reference case, with the 1.0 wt.% and 2.0 wt.% filler loading exhibiting the highest values, among others. For the 4.0 wt.% filler fraction, storage modulus at low temperatures diminished, compared to 1.0 wt.% and 2.0 wt.%, respectively, exhibiting material brittleness due to high filler inclusion. The above corroborates the findings of the static mechanical tests.

With respect to damping, tanδ curves revealed a generally similar trend, that is an increasing tendency that loses intensity at approximately 70 °C. Filler loading caused a damping effect of similar fashion to storage modulus, Tanδ values exhibited a peak value around the temperature value of 90 °C, whilst above 110 °C, tanδ values started to drop, reaching their lowest value, at approximately 120 °C. As aforementioned, an oscillating trend is observed at high temperatures that corresponds to a softening effect.

### 4.3. Spectroscopic and Microstructural Analysis

For all nanocomposites, characteristic PP Raman peaks could be seen, as has been reported elsewhere [39]. More specific, the characteristic fingerprints of PP are located at ca. 385, 810, 868, 967, 1036, 1168 and 1221 cm^−1^ (C–C stretching vibration), 1250 and 1320 cm^−1^ (CH deformation vibration), 1334 and 1454 cm^−1^ (–CH_2_ of the PP backbone macromolecular chains), 1361–1385 cm^−1^ (–CH_3_ deformation vibrations of PP chains, as well as the –CH_3_ side group rocking vibration) [39], and 2721, 2837, 2875 and 2962 cm^−1^ (CH_3_ symmetric and asymmetric stretching vibration) [40]. The spectra of PP/SiO_2_ nanocomposites at the different SiO_2_ wt.% filler loading exhibit some peaks attributed to the incorporated SiO_2_ NP vibrational modes, indicated more clearly in Figure 7b. Namely, peaks at ca. 363 cm^−1^, 451 cm^−1^, 582 cm^−1^ and 609 cm^−1^ correspond to SiO_2_ NPs, being in good agreement with the typical SiO_2_ NP vibrational modes reported elsewhere [41]. It is worth mentioning and it can be observed that the corresponding SiO_2_ NP peaks’ intensity is slightly increasing with the increased SiO_2_ wt.% filler loading.

SEM was utilized to characterize the external microstructural characteristics of the specimens, as well as the fractured surfaces of tensile test specimens also elucidating the internal structure of the 3D printed specimens. As shown in Figure 8, a high-quality 3D printing process is validated by the good interlayer fusion visible through the side captures. Also, layer height seems to be perfectly in accordance with the settings specified in the FFF process, at approximately 200 microns. Some slight defects are shown in PP/SiO_2_ 4.0 wt.%, which were possibly generated due to high filler concentration.

Adequate 3D printing, and indirectly extrusion, processes, are validated through the fracture area SEM micrographs. Visible gaps on the fractured surface are common case in 3D printed thermoplastic structures. Even 100% infill setup most times is not enough to minimize an anisotropic behavior of 3D printed parts. In the current study, both PP/SiO_2_ 0.5 wt.% (Figure 9a,b) and 2.0 wt.% (Figure 9c,d) fractured areas reveal that the 3D printed nanocomposite objects are almost perfectly fused. PP/SiO_2_ 4.0 wt.% fractured surface slightly differs (Figure 9e,f), as fusion between shells and infill of 3D printed specimen seems to be slightly worse. This can possibly be justified by the increase in the viscosity of the polymer melt, due to the increase of the silica concentration that hinders the PP macromolecular chains mobility in the melt state. It should be mentioned that this change in flow ratio did not cause any challenges during the extrusion or the 3D printing manufacturing process. Considering the surface micrographs and the fractured areas of the specimens, it could be postulated that no intense agglomeration occurred even at the highest filler loading i.e., the 4.0 wt.%, and high quality SiO_2_ NP dispersion in the PP matrix was achieved.

Regarding the extruded filament surface topography revealed in the AFM images, increasing silica loading in the PP led to a rather high effect in the filament’s surface roughness. Almost 1 μm roughness was measured for the case of PP/SiO_2_ 4.0 wt.% filament, which is almost 65% higher than the roughness of the PP/SiO_2_ 2.0 wt.% filament.

### 4.4. Melt Flow Index Analysis

MFI analysis was conducted to quantify the effect of filler loading on the rheology of the studied polymer. MVR indicates the ease of flow, which is a crucial factor for extrusion in melt-mixing and for the FFF manufacturing procedure. As aforementioned, and shown in Figure 11b, filler loading caused a lowering effect on MVR values, which denotes increase in viscosity. Increase in viscosity is an expected outcome that is attributed to the presence of particles within the polymer melt. Specifically, a drop of approximately 20% was exhibited for the case of PP/SiO_2_ 4.0 wt.% compared to neat PP. Interesting enough, after a first recorded drop of MVR values at 0.5 wt.% filler loading, MVR values exhibited a slight increase for the cases of 1.0wt.% and 2.0 wt.%, respectively, before lessening to the minimum MVR value, for the case of 4.0 wt.%. This successive alteration of MVR values was also observed for the static and viscoelastic response of the material to filler loading.

## 5. Conclusions

In this study, the effect of SiO_2_ filler loading on the mechanical, viscoelastic, rheological, and microstructural properties of isotactic PP homopolymer was studied. Silica loading appears to affect the mechanical behavior of the matrix material, even at low concentrations. Highest values of the tensile modulus of elasticity were recorded at PP/SiO_2_ 4.0 wt.%, while nanocomposites of 2.0 wt.% and 1.0 wt.% developed a similar behavior. Flexural strength follows a similar trend, while in this case PP/SiO_2_ 1.0 wt.% exhibits the highest values, also with low differences to 4.0 wt.%. Impact strength and microhardness were marginally affected by the SiO_2_ additive in the PP polymer matrix material. So, the introduction of silica to the PP polymer enhances its mechanical response in all concentrations studied.

DMA testing revealed a stiff behavior for PP/SiO_2_ 1.0 wt.% compared to other cases, which is in accordance with flexure testing. Finally, in total an improved performance is reported when using silicon dioxide as a filler material additive melt mixed with polypropylene. Specifically, an addition of 1.0–2.0 wt.% leads to improved mechanical behavior, without compromising processability of the material. SEM images from the side of the tensile test specimens showed that the specimens manufactured with the process followed in this work are of high-quality, ensuring that the process itself is reliable to be adapted in industrial environments and the quality of the specimens did not affect the results of the mechanical tests performed. Possible defects and discontinuities in the interlayer fusion could affect the mechanical properties of the specimens tested, due to low cohesion between the specimens’ strands. SEM images from the fracture surface of the tensile test specimens revealed an expected behavior for 3D printed specimens, with the development of visible gaps between the strands at failure. The increase of the viscosity at the highest filler loading studied, seems to have an effect on these phenomena on the fracture area of the specimen, since the fusion between the strand was found to be worsen in this case.

PP enhanced with silica shows a potential for applications in FFF AM technology as it has been found to be quite promising in increasing the mechanical and the dynamic thermomechanical properties of PP, as well as SiO_2_ nanocomposites was found to counterbalance wrapping effect during 3D printing, without compromising the 3D printing extrusion filamentous processing/deposition.

## Figures and Tables

**Table 1:** Summarizing of mechanical properties measured and/or calculated through tests conducted.

Silicon Dioxide Percentage (wt.%)					
Mechanical property	0	0.5	1	2	4
Tensile strength (MPa)	32.22	34.99	33.86	34.83	31.32
Tensile Modulus of elasticity (MPa)	138.21	139.59	139.04	140.01	148.58
Flexural strength (MPa)	41.54	40.13	47.11	46.69	43.28
Flexural Modulus of elasticity (MPa)	815.11	785.77	926.78	921.89	849.34
Impact Resistance (kJ/m^2^)	3.72	3.94	4.01	3.79	3.85
Microhardness (HV)	12.60	12.71	12.75	12.44	13.34

## Data Availability

The data presented in this study are available on request from the corresponding author.
